# MicroRNA‐215‐5p promotes proliferation, invasion, and inhibits apoptosis in liposarcoma cells by targeting MDM2

**DOI:** 10.1002/cam4.5993

**Published:** 2023-05-03

**Authors:** Zhengnan Song, Jingping Bai, Renbing Jiang, Junshen Wu, Wenpeng Yang

**Affiliations:** ^1^ Department of Bone and Soft Tissue Xinjiang Tumor Hospital Urumqi China

**Keywords:** apoptosis, invasion, liposarcoma, *MDM2*, miR‐215‐5p, proliferation

## Abstract

**Background:**

Liposarcoma (LPS) is one of the most common soft tissue malignancies in adults, and it is characterized by dysregulation of multiple signaling pathways, including MDM2 proto‐oncogene (MDM2) amplification. MicroRNA (miRNA) regulates gene expression through incomplete complementary pairing with the 3' untranslated region of mRNAs involved in tumor progression.

**Methods:**

In this study, bioinformatics analysis, RT‐qPCR, dual‐luciferase reporter gene, MTT, flow cytometry, cell scratches, chamber migration, colony formation, FISH, WB, and CCK8 were used.

**Results:**

RT‐qPCR showed that the expression of MDM2 was increased when miR‐215‐5p was overexpressed compared with the control group. The dual‐luciferase reporter gene showed that the Renilla ratio firefly fluorescence intensity was decreased in the overexpression group compared with the control group. Cell phenotype experiments revealed that the overexpression group had increased cell proliferation rate, increased apoptosis rate, increased colony formation rate, increased cell healing area ratio, and increased number of cell invasions. FISH revealed increased MDM2 expression in the overexpression group. WB suggested decreased Bax expression, increased PCNA, Bcl‐2, and MDM2 expression, and decreased P53 and P21 expression in the overexpression group.

**Conclusions:**

In this study, we suggest that miR‐215‐5p can target and promote MDM2 expression, promote the proliferation and invasion of LPS cells SW‐872, and inhibit apoptosis.Targeting miR‐215‐5p may be a novel therapeutic strategy for the treatment of LPS.

## 1 INTRODUCTION

Soft tissue malignancies (STS) are defined as a group of malignancies derived from non‐epithelial extra osseous tissues and are a highly heterogeneous group of tumors.^1^ STS accounts for approximately 1% of all malignancies in humans,^2^ with an annual incidence of approximately 3.4 per 100,000 in the United States^3^ and 5 per 100,000 in Europe.^4^ The incidence of STS has gradually increased in recent years and has increased more significantly with age.^5^ Liposarcoma (LPS) is one of the major types of STS in adults, accounting for approximately 15%–20% of STS in adults.^5^ LPS often starts by gradually growing painless mass and the prognosis is correlated with the site of growth and the degree of malignancy.^6^ The evaluated extended surgical resection can significantly improve the survival of LPS patients according to the indications and it is the preferred regimen for LPS therapy.^7^ LPS are locally aggressive, show infiltration growth or destructive growth and are prone to local recurrence and distant metastasis and the 5‐year overall survival rate of LPS with regional or distant metastasis is less than 50%.^8^ LPS mainly includes four subtypes, well differentiated (WDL), dedifferentiated (DDL), myxoid (MLS), and pleomorphic (PLS), of which DDL have the highest malignancy with a 10‐year survival rate of less than 10%.^8^ Benefiting from the development of molecular pathology and the application of next‐generation sequencing, the study of molecular aberrations in LPS is more refined and opens the possibility of more precise targeted therapy.^6^

*MDM2* proto‐oncogene (*MDM2*) is currently one of the most studied targeted therapeutic sites for LPS. Aberrant amplification of *MDM2* in the genome is present in almost all WDL or DDL^9^ and this amplification is considered an important molecular event in the formation of WDL or DDL. Elevated *MDM2*, as measured by genome amplification and mRNA expression, is associated with a shorter time to relapse.^9^ It has been confirmed that high amplification of *MDM2* is associated with poor prognosis and the poor response to cytotoxic drugs.^10^, ^11^

The *MDM2* gene is located on human chromosomes 12q14.3 to q15, with a total length of 491 amino acids and responds to cellular stress stimuli in a tumor protein p53 (TP53 or P53)‐dependent manner, which is involved in cell growth inhibition, apoptosis induction and cell cycle regulation.^12^ In normal cells, *MDM2* and wild‐type *P53* regulate with each other to form a negative feedback loop. The P53 protein can bind to the P2 promoter on MDM2 and activate MDM2 expression at the transcriptional level; MDM2 protein binds to key amino acid residues of the N‐terminal transactivation domain of P53 and forms a P53‐MDM2 complex to inhibit the transcriptional activity of *P53*.^13^ Second, the MDM2 proteasome itself can act as an E3 ubiquitin ligase that can mediate P53 ubiquitination and degradation, allowing P53 to be maintained at normal physiologically low levels.^14^ MDM2 overexpression can downregulate P53 activity and promote P53 degradation through the proteasome pathway, which in turn leads to tumor suppressor P53 pathway inactivation, allowing cells to pass through the cell cycle in the presence of DNA damage, which in turn initiates tumorigenesis.^15^ In addition, MDM2 can regulate cell cycle and apoptosis by interacting with tumor growth suppressors other than P53.^16^ Cyclin‐dependent kinase inhibitor 1A (CDKN1A or P21) is a downstream gene of P53, which encodes a protein that can arrest cell cycle progression and promote apoptosis^17^ and MDM2 can promote its degradation by ubiquitinating the marker P21 protein and maintaining a low‐level state of P21 in the absence of stress signals.^18^ An critical factor in the abnormal expression of MDM2 in LPS is the disturbance of the regulation of MDM2 expression and the study of the regulatory mechanism of MDM2 is helpful to further explore the pathogenesis of LPS and explore new targeted therapeutic sites.

MDM2 inhibitors can block the interaction between MDM2 and P53 and upregulate P53 and P21 expression, which in turn inhibits tumor cell proliferation and promotes apoptosis.^16^ Phase I clinical studies showed that after treatment with the MDM2 inhibitor SAR405838 in LPS patients with MDM2 amplification, 71% of patients achieved SD.^19^ MDM2 inhibitors are an important research direction of LPS targeted therapy,^20^ and the molecular mechanism of MDM2 expression regulation also provides some theoretical reference for the optimization and improvement of MDM2 inhibitors.

MiRNAs are non‐coding RNAs of approximately 19–25 nucleotides in length and MiRNAs can promote or inhibit target gene expression at the translational level through incomplete pairing with the three ‘untranslated regions of target genes,^21^ which in turn repress target gene expression at the translational level. The regulation of gene expression by miRNAs can oscillate between inhibition and stimulation in response to specific cellular conditions, sequences, and co‐factors. Recent emerging evidence suggests that miRNAs can mediate upregulation of gene expression.^22^ For example, Zhen^23^ and others found that in cervical cancer cells, MIR‐G‐1 can directly target the 3 ‘UTR that binds transmembrane p24 trafficking protein 5 (TMED5) and lamin B1 (LMNB1) and promote their expression, which in turn promotes the proliferation, migration, and invasion of malignant cells in cervical cancer. MiRNAs are involved in the regulation of various intracellular biological processes and more than one‐third of human genes are targeted and regulated by miRNAs. Precise control of miRNA levels is essential for maintaining normal cellular function, while abnormal levels of intracellular miRNAs are closely related to the process of tumorigenesis and progression^24^; so miRNAs are promising as predictive biomarkers for the diagnosis, prognosis, and possible therapeutic targets of tumors.^24^

In summary, MDM2 is an important oncogene and targeted therapeutic site in LPS and MIRNA is an important regulatory molecule for gene expression. At present, miRNAs related to MDM2 regulation in LPS have not been fully clarified and corresponding experimental evidence is lacking. In our study, we selected miR‐215‐5p that may target the MDM2 gene. The purpose of our study was to investigate the effect of miR‐215‐5p overexpression and underexpression on MDM2 expression in LPS cells and whether there is a direct regulatory relationship between the two. We expected to demonstrate the effect of miR‐215‐5p overexpression and low expression on biological phenotypes such as LPS cell proliferation activity, apoptosis, cell cycle progression, cell invasiveness, and metastasis by cell platform. We also expect to confirm the expression changes of caspase‐3, MDM2, P53, P21, proliferating cell nuclear antigen (PCNA), BCL2‐associated X (Bax), and BCL2 apoptosis regulator (Bcl‐2) when miR‐215‐5p is overexpressed and lowly expressed, as well as to add MDM2 inhibitors to further explore the molecular mechanism of miR‐215‐5p regulation. We hope to further explore the regulatory mechanism associated with MDM2 expression in LPS and provide some theoretical supplement for targeting MDM2 in the treatment of LPS; by exploring the function of miR‐215‐5p in LPS, we explore the possibility of phase as a new targeted therapeutic site.

## 2 RESULTS

### 2.1 miRNA and gene selection

**FIGURE 1:**
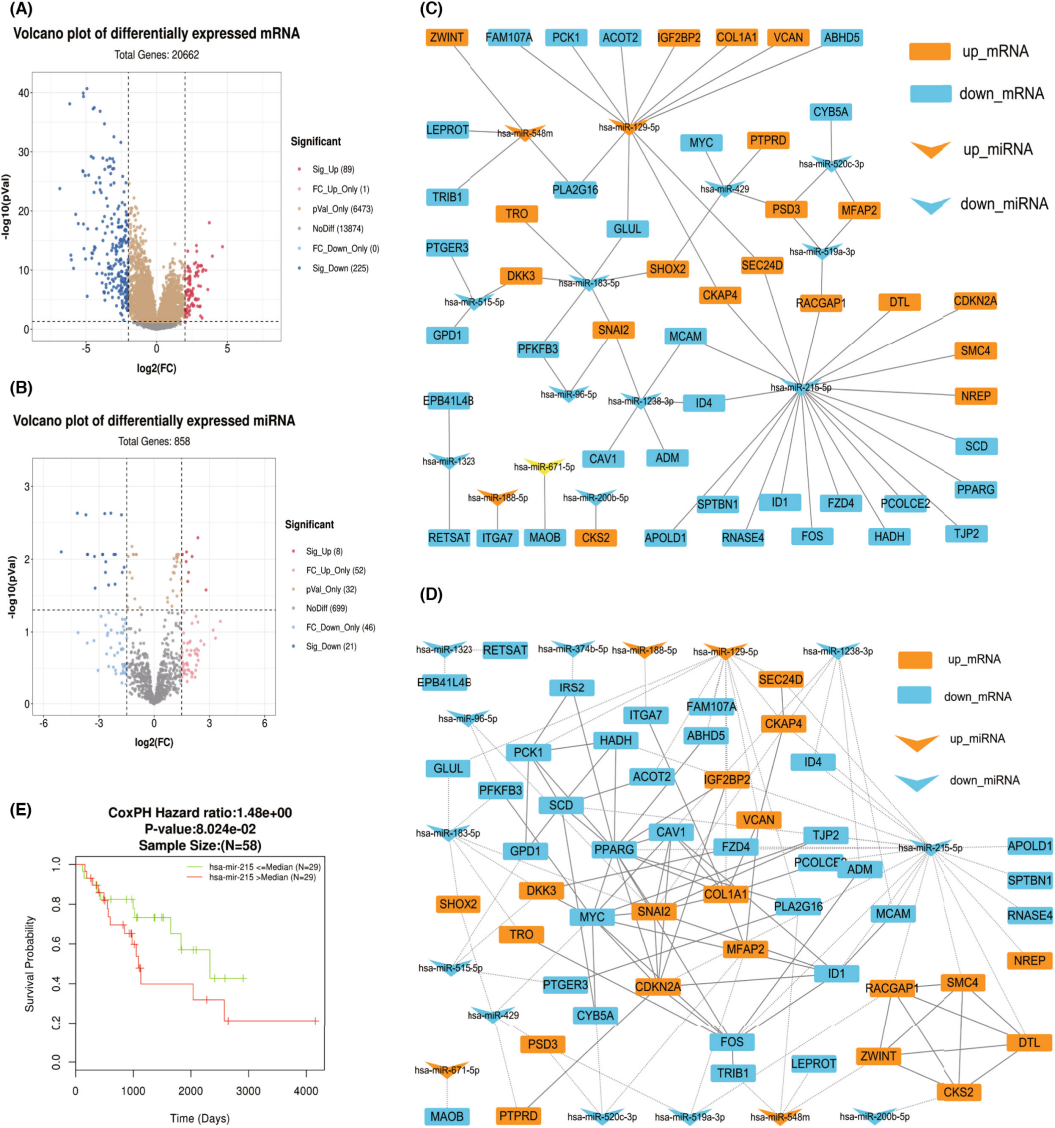
miRNA and gene selection. A and B screened differentially expressed mRNAs (A) and miRNAs (B) between LPS tissues and normal tissues; (C) shows the experimentally confirmed regulatory relationship between these mRNAs and miRNAs; (D) shows the miRNA–mRNA regulation and protein interaction fusion network, in which miR‐215‐5p is in the central regulatory position; (E) shows the survival analysis of the high and low miR‐215‐5p expression groups in LPS of TCGA.

We screened differentially expressed mRNA (Figure 1A) and miRNA (Figure 1B) information between LPS and normal control tissues from the GEO database and performed GO (Supplement 1A) and KEGG (Supplement 1B) functional enrichment analysis of differentially expressed genes. We predict all regulatory target genes of differentially expressed miRNAs and construct mRNA‐miRNA regulatory networks (Figure 1C) by overlapping genes of differentially expressed miRNA target genes with differentially expressed mRNAs. We applied STRING to construct a differentially expressed protein–protein interaction network and fused it with the miRNA–mRNA regulatory network (Figure 1D) and topologically analyzed the fusion network to screen the top 10 key nodes (Table 1), which included one miRNA, miR‐215‐5p. For the survival analysis of DDL from the TCGA database (Figure 1E), miR‐215‐5p was suggested to be associated with poor long‐term prognosis, although *p* = 0.08. At last, we used TargetScan to predict miR‐215‐5p in relation to *MDM2* regulation (Table 2). Combined with the published literature, we predict that there is a possible regulatory relationship between miR‐215‐5p and *MDM2* in LPS.

**TABLE 1:** Top 10 key nodes in network.

Rank	Name	Score
1	CDKN2A	75
2	MYC	66
3	PPARG	60
3	SNAI2	60
5	CAV1	56
6	ID1	38
7	miR‐215‐5p	36
8	RACGAP1	31
9	DTL	30
9	SMC4	30

**TABLE 2:** TargetScan prediction results.

Seed of miR‐215‐5p																															Site type	Contex++Score
Position 4592–4598 of MDM2 3’ UTR	5’		C	U	C	C	C	U	G	U	C	U	U	C	U	C	U	U	A	G	G	U	C	A	C						poorly conserved 7‐mer‐m8	−0.08
																|	|	|	|	|												
miR‐215‐5p	3’									C	A	G	A	C	A	G	U	U	A	A	G	U	A	U	C	C	A	G	U	A		

### 2.2 Induction of miR‐215‐5p expression in LPS cells

**FIGURE 2:**
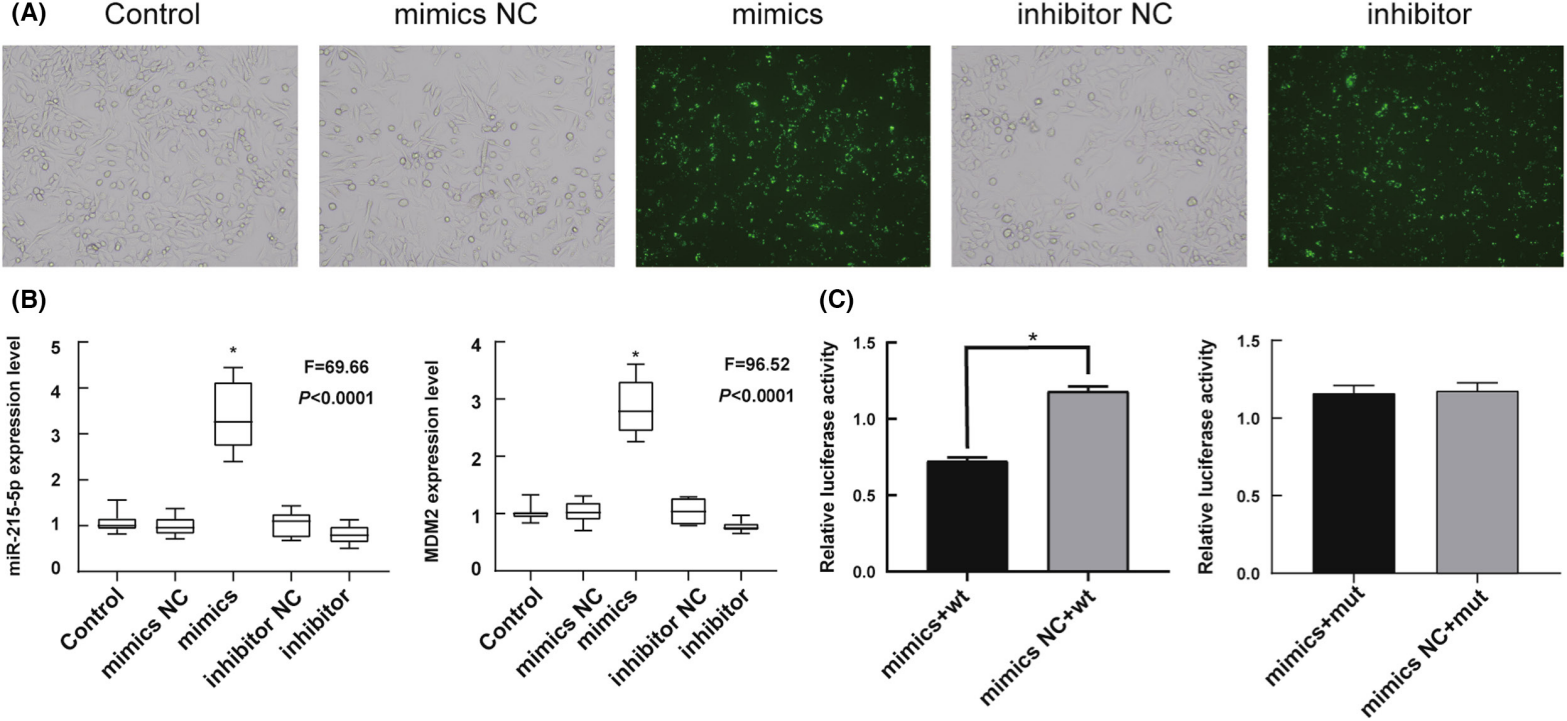
miR‐215‐5p directly promotes MDM2 expression in LPS cells. (A) Shows that after miR‐215‐5p mimcs and miR‐215‐5p inhibitor transfection of cells, green fluorescence appeared; 100X magnification; (B) indicates that the expression of miR‐215‐5p and MDM2 is significantly increased in the mimics group compared with the control group; (C) shows that co‐transfer of miR‐215‐5p‐mimics + wt plasmid results in a decrease in the ratio of Renilla specific firefly fluorescence intensity compared with the control group, and there is no significant difference in the others. *p < 0.05.

The plasmid containing miR‐215‐5p was transferred into human LPS cells SW‐875. Successful transfection was judged by observing miR‐215‐5p transfection into SW‐872 cells. After transfection of cells with miR‐215‐5p mimics and miR‐215‐5p inhibitor, green fluorescence appeared (Figure 2A) and transfection was successful. The transfection effect was detected by real‐time PCR of transfected cells. The experimental results indicated that the expression of miR‐215‐5p in the miR‐215‐5p mimics group was 3.37 ± 0.74, which was significantly increased compared with 1.07 ± 0.22 in the control group, *p* < 0.05 (Figure 2B).

### 2.3 *MDM2* expression in LPS cells

Expression of the *MDM2* gene was analyzed by RT‐qPCR after confirming significant overexpression of miR‐215‐5p in the transduced cell lines. We found that the expression of *MDM2* was 2.85 ± 0.48, which was significantly increased compared with 1.00 ± 0.14 in the control group, *p* < 0.05 (Figure 2B).

### 2.4 miR‐215‐5p directly regulates *MDM2* expression

The relationship between miR‐215‐5p and the target gene *MDM2* was examined using a dual‐luciferase reporter gene. The experimental results showed that co‐transfer of miR‐215‐5p‐mimics + wt plasmid resulted in a fluorescence intensity ratio of 0.72 ± 0.02 obtained by Renilla luciferase/firefly luciferase assay, which was significantly lower than the fluorescence intensity ratio of 1.18 ± 0.03 co‐transferred with miR‐215‐5p‐mimics NC + wt plasmid, *p* < 0.05. There was no significant difference between the other groups, *p* > 0.05 (Figure 2C).

### 2.5 miR‐215‐5p promotes proliferation of SW‐872 cells

**FIGURE 3:**
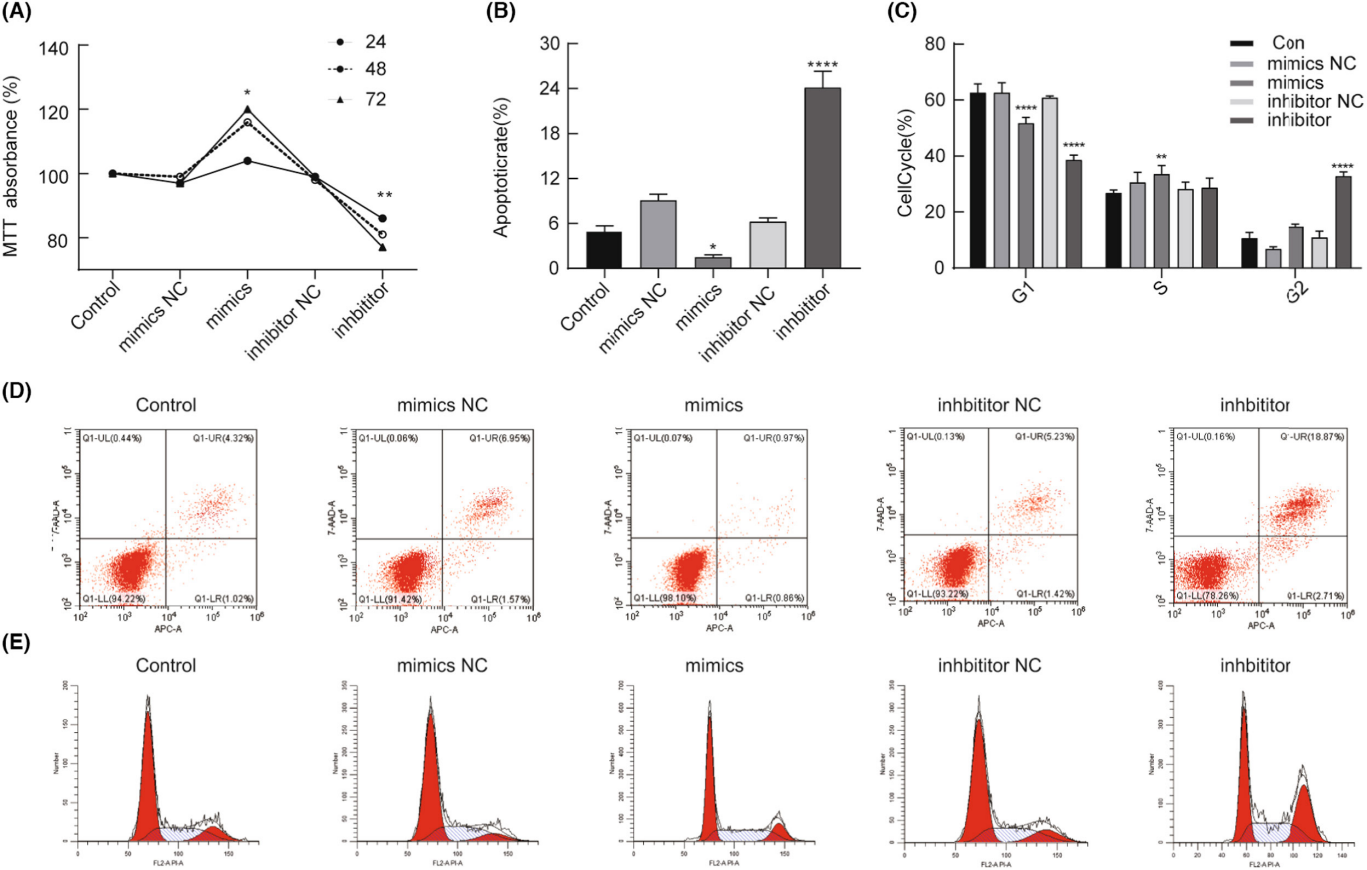
miR‐215‐5p promotes proliferation and inhibits apoptosis in LPS cells. (A) Indicates that the cell proliferation rate in the miR‐215‐5p mimics group is significantly increased compared with the control group; (B) indicates that the apoptosis rate of the mimics group is significantly decreased compared with the control group, and the apoptosis rate of the inhibitor group is significantly increased compared with the control group; (C) indicates that the cell cycle of the mimics group is significantly decreased in G1 phase, significantly increased in S phase, and did not change significantly in G2 phase, and the inhibitor group is significantly decreased in G1 phase, not significantly different in S phase, and significantly increased in G2 phase compared with the control group;(D) is the raw data plot of apoptosis detected by flow cytometry; (E) is the original figure of flow cytometric cycle. *p < 0.05, **p < 0.01, ****p < 0.001.

We investigated the functional effects of miR‐215‐5p mimics and inhibitor on SW‐872 cells by MTT colorimetric assay. The experimental results revealed that the cell proliferation rates of miR‐215‐5p mimics at 24, 48, and 72 h were 104 ± 0.04%, 116 ± 0.01%, and 120 ± 0.01%, respectively, which were significantly higher than those of the control group (100 ± 0.00%, *p* < 0.05); while the cell proliferation rates of miR‐215‐5p inhibitor at 24, 48, and 72 h were 86 ± 0.01%, 81 ± 0.01%, and 77 ± 0.01%, respectively, which were significantly lower than those of the control group (100 ± 0.00%, *p* < 0.05) (Figure 3A). The results confirmed that miR‐215‐5p significantly promoted cell proliferation.

### 2.6 miR‐215‐5p inhibits apoptosis

We analyzed the effects of miR‐215‐5p on apoptosis and cell cycle in SW‐872 cells by flow cytometry. The results showed that the apoptosis rate of the miR‐215‐5p mimics group was 1.47 ± 0.33%, which was significantly lower than that of the control group (4.89 ± 0.79%, *p* < 0.05); the apoptosis rate of the miR‐215‐5p inhibitor group was 24.12 ± 2.20%, which was significantly higher than that of the control group (4.89 ± 0.79%, *p* < 0.05) (Figure 3B). Figure 3D shows the raw data plots for flow cytometric apoptosis.

### 2.7 miR‐215‐5p promotes cell cycle progression

The cell cycle of the miR‐215‐5p mimics group was 51.79 ± 2.05% in G1 phase, which was significantly lower than 62.65 ± 3.12% in the control group, *p* < 0.05; 33.54 ± 3.10% in S phase was significantly higher than 26.75 ± 1.12% in the control group, which did not change significantly in G2 phase, *p* > 0.05. The cell cycle of the miR‐215‐5p inhibitor group was 38.57 ± 1.82% in G1 phase, which was significantly lower than 62.65 ± 3.12% in the control group, *p* < 0.05; there was no significant difference in S phase, *p* > 0.05; and 32.74 ± 1.62% in G2 phase was significantly higher than 10.61 ± 2.09% in the control group, *p* < 0.05 (Figure 3C). Figure 3E shows the raw data plots for the flow cytometric cycle.

### 2.8 miR‐215‐5p promotes invasion and metastasis of SW‐872 cells

**FIGURE 4:**
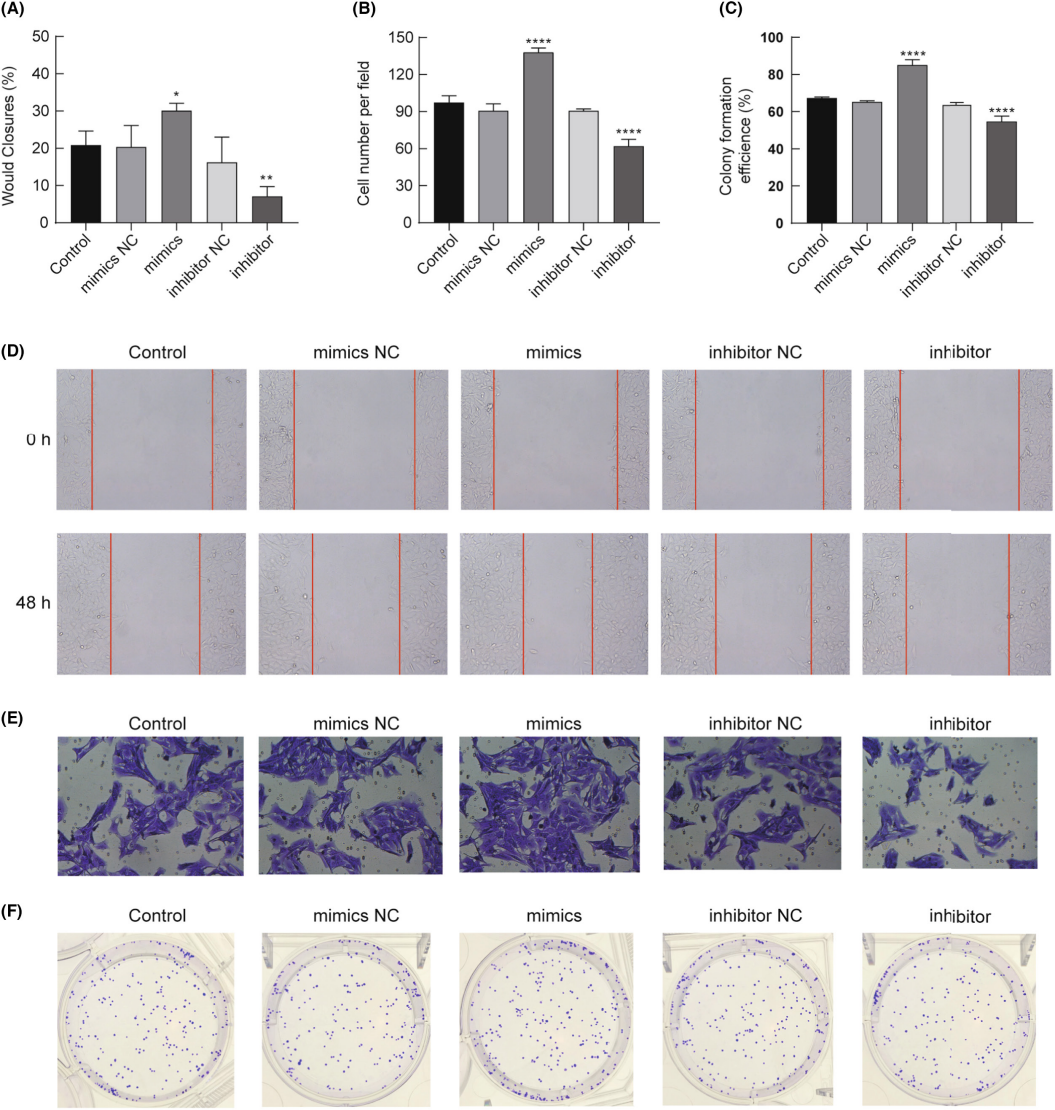
miR‐215‐5p promotes invasion and metastasis in LPS cells. (A) indicates that the cell healing area ratio is significantly increased in the mimics group and significantly decreased in the inhibitor group compared with the control group; (B) indicates that the number of cell invasions is significantly increased in the mimics group and significantly decreased in the inhibitor group compared with the control group; (C) indicates that the colony formation rate of the mimics group is significantly increased, and the colony formation of the inhibitor group was significantly decreased compared with the control group; (D) is the original figure of cell healing assay, 100× magnification; (E) is the original figure of cell invasion assay, 100× magnification; and (F) is the original figure of the cell colony formation assay. *p < 0.05, **p < 0.01, ****p < 0.001.

We investigated the effects of miR‐215‐5p mimics and inhibitor on the invasion and healing ability of SW‐872 cells by cell scratch and invasion assays. The experimental results revealed that the cell healing area ratio of the miR‐215‐5p mimics group was 30.08 ± 2.00%, which was significantly higher than that of the control group 20.86 ± 3.77%, *p* < 0.05; the cell healing area ratio of the miR‐215‐5p inhibitor group was 7.12 ± 2.5%, which was significantly lower than that of the control group 20.86 ± 3.77%, *p* < 0.05 (Figure 4A). Figure 4D shows the original images of cell scratches.

The number of cell invasion in the miR‐215‐5p mimics group was 138 ± 3.60, which was significantly increased compared with 97.33 ± 5.51 in the control group, *p* < 0.05; the number of cell invasion in the miR‐215‐5p inhibitor group was 62 ± 5.57, which was significantly decreased compared with 97.33 ± 5.51 in the control group, *p* < 0.05 (Figure 4B). Figure 4E shows the original images of cell invasion.

Colony formation in the miR‐215‐5p mimics group was 85.10 ± 2.85%, which was significantly higher than that in the control group 67.33 ± 0.58%, *p* < 0.05; colony formation in the miR‐215‐5p inhibitor group was 54.57 ± 3.01%, which was significantly lower than that in the control group 67.33 ± 0.58%, *p* < 0.05 (Figure 4C). Figure 4F shows the original images of cell clones.

### 2.9 miR‐215‐5p promotes MDM2 expression

**FIGURE 5:**
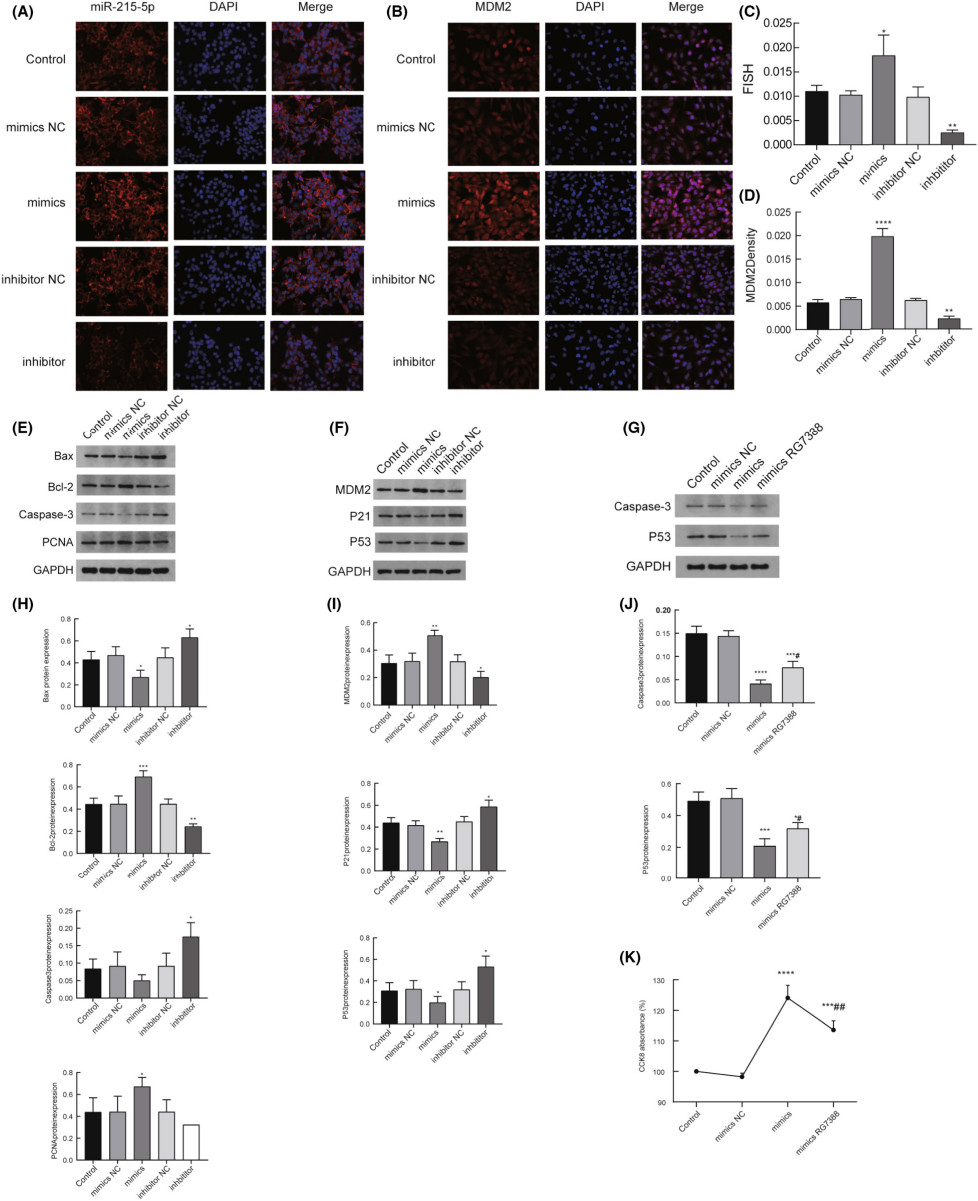
miR‐215‐5p effects on gene expression. (A) indicates the localization of MDM2 and miR‐215‐5p, 400× magnification; (B) represents MDM2 detected by immunofluorescence single labeling assay, 400× magnification; (C) and (D) indicate that the expression of overexpressed MDM2 is significantly increased, and the expression of inhibited MDM2 was significantly decreased compared with the control group; (E) and (F) represent the original figures of the expression changes of caspase‐3, MDM2, p53, P21, PCNA, bax, and bcl‐2 detected by WB; (G) indicates that miR‐215‐5p mimics are added with MDM2 inhibitor RG7388, the expression changes of caspase 3 and P53 are detected by WB; (H) indicates that compared with the control group, the expression of PCNA and Bcl‐2 is significantly increased, the expression of Bax is significantly decreased, and the expression of caspase‐3 is not statistically significant, while the expression of PCNA is not statistically significant, the expression of Bcl‐2 is significantly decreased, and the expression of Bax and caspase‐3 is significantly increased in the overexpression group; (I) indicates that the expression of MDM2 is significantly increased and the expression of P53 and P21 is significantly decreased in the overexpression group, while the expression of MDM2 is significantly decreased and the expression of P53 and P21 is significantly increased in the inhibition group, compared with the control group; (J) shows that by adding the MDM2 inhibitor RG7388 to the mimic, the expression of caspase3 is significantly increased compared with the mimic group, but still decreased compared with the control group, and the expression of P53 is significantly increased compared with the mimic group, but still decreased compared with the control group; (K) indicates that the cell proliferation activity is significantly lower than that in the mimics group after the addition of inhibitors but still increased compared with the control group.

In this study, we detected the localization and expression of miR‐215‐5p and *MDM2* by FISH. The expression level of *MDM2* in the miR‐215‐5p mimics group was 0.02 ± 0.002, which was significantly higher than that of the control group (0.006 ± 0.0007), *p* < 0.05 (Figure 5C); the expression level of *MDM2* in the miR‐215‐5p inhibitor group was 0.002 ± 0.0005, which was significantly lower than that of the control group (0.006 ± 0.0007), *p* < 0.05 (Figure 5D). Figure 5A was FISH raw plot for miR‐215‐5p and Figure 5B was FISH raw plot for *MDM2*.

### 2.10 miR‐215‐5p promotes cell proliferation and inhibits apoptosis

We found by western blot experiments that the expression of PCNA in the miR‐215‐5p overexpression group was 0.672 ± 0.08, which was significantly higher than that in the control group 0.44 ± 0.13, *p* < 0.05; the expression of PCNA after inhibition of miR‐215‐5p was 0.32 ± 0.15, which was not significantly different from that in the control group 0.44 ± 0.13, *p* > 0.05. At the same time, the expression of Bcl‐2 by miR‐215‐5p overexpression was 0.69 ± 0.05, which was significantly increased compared with 0.44 ± 0.05 in the control group, *p* < 0.05; the expression of Bax was 0.27 ± 0.06, which was significantly decreased compared with 0.43 ± 0.07 in the control group, *p* < 0.05; and the expression of caspase‐3 was 0.05 ± 0.02, which was not significantly different from 0.08 ± 0.03 in the control group, *p* > 0.05.

After inhibition of miR‐215‐5p, the expression of Bcl‐2 was 0.24 ± 0.02, which was significantly lower than 0.44 ± 0.05 in the control group, *p* < 0.05; the expression of Bax was 0.63 ± 0.08, which was significantly higher than 0.43 ± 0.07 in the control group, *p* < 0.05; and the expression of Caspase‐3 was 0.18 ± 0.04, which was significantly higher than 0.08 ± 0.03 in the control group, *p* < 0.05 (Figure 5H). Figure 5E is the original plot of this part WB and GAPDH is the control.

### 2.11 miR‐215‐5p promotes the proliferation of cells by targeting MDM2

Western blot showed that the expression of MDM2 was 0.51 ± 0.04 in the miR‐215‐5p overexpression group, which was significantly higher than 0.30 ± 0.06 in the control group, *p* < 0.05; after inhibition of miR‐215‐5p, the expression of MDM2 was 0.20 ± 0.04, which was significantly lower than 0.30 ± 0.06 in the control group, *p* < 0.05. For the miR‐215‐5p overexpression group, the expression of P53 was 0.20 ± 0.06, which was significantly lower than 0.31 ± 0.08 in the control group, *p* < 0.05; after inhibition of miR‐215‐5p, the expression of P53 was 0.53 ± 0.10, which was significantly higher than 0.31 ± 0.08 in the control group, *p* < 0.05. For miR‐215‐5p overexpression, the expression of P21 was 0.27 ± 0.03, which was significantly lower than 0.44 ± 0.05 in the control group, *p* < 0.05; after inhibition of miR‐215‐5p, the expression of P21 was 0.59 ± 0.10, which was significantly higher than 0.44 ± 0.05 in the control group, *p* < 0.05 (Figure 5I). Figure 5F is the original plot for WB and GAPDH is the control.

### 2.12 MDM2 inhibitors can restore P53 activity and cell proliferation

By adding MDM2 inhibitor RG7388 to miR‐215‐5p mimics, we found that the protein expression of Caspase‐3 was 0.08 ± 0.01 after adding the inhibitor, which was significantly higher than 0.04 ± 0.01 in the mimics group, *p* < 0.05, but still decreased compared with 0.15 ± 0.02 in the control group, *p* < 0.05; the protein expression of P53 was 0.32 ± 0.04, which was significantly higher than 0.21 ± 0.04 in the mimics group, *p* < 0.05, but still decreased compared with 0.49 ± 0.06 in the control group, *p* < 0.05 (Figure 5J) and after adding the inhibitor group, the cell proliferation activity was 113.60 ± 3.00%, which was significantly lower than 124.10 ± 4.10% in the mimics group, *p* < 0.05, but still increased compared with 100.00 ± 0.00% in the control group, *p* < 0.05 (Figure 5K). Figure 5G is the original plot of this part WB and GAPDH is the control. It was also shown to some extent that miR‐215‐5p exerts biological functions by targeting MDM2, which begins to return to normal when MDM2 inhibitors are added.

## 3 DISCUSSION

In this study, we found a binding pairing relationship between miR‐215‐5p and MDM2 3 ‘UTR and MDM2 expression was activated when miR‐215‐5p was overexpressed. MDM2 is one of the most studied targeted therapeutic sites of LPS in recent years^9^ and is the main negative regulator of P53.^12^ MDM2, which is overexpressed in LPS, can inhibit P53 transcriptional activity and promote P53 degradation, leading to inactivation of the tumor‐suppressive P53 pathway, thereby promoting LPS pathogenesis and progression.^12^ MDM2 inhibitors can block the interaction between MDM2 and P53^13^ and then upregulate the expression levels of P53 and its downstream P21,^14^ exerting the effects of inhibiting LPS cell proliferation and promoting apoptosis. In this study, we predicted that miR‐215‐5p may target and regulate MDM2 in LPS and the results of bioinformatics analysis suggested that miR‐215‐5p was downregulated in LPS, which was consistent with the results that microarray technology revealed that miR‐215‐5p was downregulated in LPS.^25^

MiRNAs function mainly by mediating gene silencing at three stages: pretranslational,^26^, ^27^ co‐translational,^28^ and posttranslational,^29^ with the most common regulatory mechanism being incomplete complementary pairing with 3 ‘UTR sites in target mRNAs.^19^ In addition, some miRNAs is targeted to the mRNA 5 ′ UTR or coding region to regulate expression.^30^, ^31^ Some emerging evidence suggests that miRNA regulation of gene expression can oscillate between inhibition and stimulation in response to specific cellular conditions, sequences, and cofactors.^21^ Evidence for miRNA‐mediated upregulation of gene expression such as Zhen^22^ found that MIR‐G‐1 can directly target the 3 ‘UTR that binds TMED5 and LMNB1 in cervical cancer cells and promote their expression, which in turn promotes cervical cancer cell proliferation, migration, and invasion. One possible mechanism of miRNA‐mediated upregulation of gene expression is through competition with AU‐rich element (are‐)‐mediated decay pathways,^32^ AU‐rich mRNAs in 3 ‘UTRs can be degraded by are‐binding proteins (ABPs) in the cytoplasm,^33^ while if miRNAs share binding sites with the ARE‐mediated decay pathway in mRNA 3’ UTRs, they can lead to increased target mRNA stability preventing ABPs association to inhibit their degradation. In addition, miR‐744 may upregulate CCNB1 expression by promoting enrichment of RNA polymerase II and methylation of histone 3 at lysine at the CCNB1 transcription start site.^34^ MiR‐122 can enhance gene replication by targeting 5‐non‐coding elements in the hepatitis C virus genome.^35^ In addition, G‐rich RNA sequence‐binding factor 1 can upregulate TERT/hTERT by directly binding to the miR‐346 sequence, which in turn promotes TERT mRNA recruitment to ribosomes to promote translation.^36^ Another way by which miRNAs upregulate gene expression is that miRNAs located in the nucleus can bind and activate target gene enhancers and promote target gene activation at the transcriptional level.^22^

In this study, whether the regulatory mechanism of miR‐215‐5p with MDM2 is achieved by preventing ABPs association or recruiting ribosomes needs to be further investigated.

The findings presented in this study suggest that miR‐215‐5p may function as an oncogene in LPS. Although the function of miR‐215‐5p in LPS has not previously been fully clarified, it has been shown in other tumor types that miR‐215 can act as an oncogene or tumor suppressor gene. Downregulation of miR‐215 expression in colorectal cancer (CRC) promotes CRC progression and invasion by targeted inhibition of stearoyl‐CoA desaturase (SCD)^37^ and is associated with CRC malignancy.^38^ miR‐215‐5p is upregulated in multiple myeloma (MM) and low expression levels are associated with poor prognosis^39^ and upregulation of miR‐215‐5p expression can inhibit RUNX1 transcription and translation in MM, while inhibiting PI3K/AKT/mTOR pathway activation, promoting MM cell apoptosis, arresting the cell cycle in G1 phase and inhibiting cell proliferation.^39^ It has been shown that miR‐215 can also act as an oncogene in other tumors, consistent with the results of this study. Upregulation of miR‐215 expression promotes cell proliferation in high‐grade gliomas, while downregulation of miR‐215 expression inhibits cell proliferation^40^ and miR‐215 has a negative correlation with retinoblastoma 1 (RB1) and may be its regulatory target.MiR‐215 expression is upregulated in gastric cancer (GC) and correlates with tumor invasion and TNM stage and upregulation of miR‐215 expression can promote cell migration and invasion and its regulated targets are RB1,^41^ FOXO1^42^ and RUNX1.^43^ In this study, the results of cell phenotype experiments suggest that miR‐215‐5p may serve as a new targeted therapeutic site for LPS and provide some theoretical basis for targeted inhibition of miR‐215‐5p in the treatment of LPS.

P53 is the main negative regulatory target of MDM2,^44^ P21 is a downstream P53 gene^45^ and a negative regulatory target of MDM2^46^ and the protein encoded by P21 can block cell cycle progression and promote apoptosis.^47^ In this study, we suggest that miR‐215‐5p targets MDM2 in LPS and inhibits both MDM2‐regulated P53 and P21 expression, but there is insufficient evidence to indicate whether miR‐215‐5p directly regulates P53 and P21.

High PCNA expression is associated with active cell proliferation^48^ and upregulation of miR‐215‐5p was found to promote PCNA expression in this study, which suggests that upregulation of miR‐215‐5p expression in LPS can promote cell proliferation. Bcl‐2^49^ is an oncogene that inhibits apoptosis,^50^ caspase‐3^51^ is an important terminal cleaving enzyme in the process of apoptosis and Bax is one of the important pro‐apoptotic genes.^52^ In this study, we found that upregulation of miR‐215‐5p expression could significantly promote Bcl‐2 expression and significantly inhibit Bax expression; downregulation of miR‐215‐5p expression could significantly promote caspase‐3 expression and significantly promote Bax expression, suggesting that overexpression of miR‐215‐5p in LPS could significantly inhibit apoptosis, while inhibition of miR‐215‐5p expression could significantly promote apoptosis. The results of cell phenotype and WB together suggest that miR‐215‐5p can affect LPS proliferation and apoptotic function, which further suggests the theoretical possibility of targeting miR‐215‐5p to treat LPS.

The rescue assay showed that the inhibitory effect of miR‐215‐5p on P53 expression could be partially attenuated, but not completely eliminated, by adding RG7388, an MDM2 inhibitor, to the miR‐215‐5p overexpression group; the promoting effect of miR‐215‐5p on LPS cell proliferation could be partially attenuated, but not completely eliminated. The results of this biological function reversion further suggest that miR‐215‐5p acts by targeting MDM2. We speculated that the MDM2 inhibitor did not completely abolish the effect of miR‐215‐5p, suggesting that miR‐215‐5p may also affect P53 expression and LPS cell proliferation activity through other regulatory modalities, or there may be an interaction relationship with the MDM2 inhibitor.

In conclusion, we predicted the regulation of MDM2 by miR‐215‐5p in LPS by bioinformatics and experimentally demonstrated that miR‐215‐5p binds to MDM2 with a 3 ′ UTR and promotes MDM2 expression and demonstrated the function and partial mechanism of action of miR‐215‐5p in LPS cells, providing some theoretical supplements for targeting MDM2 to treat LPS and providing some theoretical basis for miR‐215‐5p as a new therapeutic target of LPS. Unfortunately, this study focused on cell experimental validation, did not use other LPS cell lines and LPS subtype cell lines as study subjects or controls and lacked animal models, which somewhat weakened the strength of the demonstration of the study results and should be supplemented in subsequent experiments; there was a lack of clinical tissue sample validation and the survival analysis results of miR‐215‐5p in TCGA were not statistically significant and further studies should supplement clinical samples and increase sample size.

## 4 CONCLUSION

In this study, we suggest that miR‐215‐5p has a critical regulatory role in LPS and can be targeted to promote MDM2 expression. miR‐215‐5p overexpression in LPS cells SW‐872 could promote cell proliferation, inhibit apoptosis, promote cell cycle progression and promote cell invasion and migration; downregulation of miR‐215‐5p expression could inhibit cell proliferation, promote apoptosis, arrest cell cycle in G2 phase, and inhibit cell invasion and migration. miR‐215‐5p may be one of the novel targeted therapeutic sites for LPS.

## 5 MATERIALS AND METHODS

### 5.1 Bioinformatic analysis

The GEO database was used for the screening of differentially expressed mRNAs and miRNAs between LPS and normal control tissues, where mRNA data were derived from GSE21122 and miRNA data were derived from GSE36982. Screening was performed by GEO2R (https://www.ncbi.nlm.nih.gov/geo/geo2r/). The screening criteria for mRNA were | logFC | > 2 and Bonferroni corrected *p* < 0.05 and the screening criteria for miRNA were | logFC | > 1.5 and Bonferroni corrected *p* < 0.05. The selected differentially expressed genes were subjected to GO and KEGG functional enrichment analysis by applying STRING and the screening criteria were taken as *p* < 0.05 after correction. MiRTarBase,^53^ miRWalk,^54^ and TargetScan^55^ databases were applied to predict all regulated target genes of differentially expressed miRNAs. Only target genes that were predicted simultaneously in the three databases were included in the analysis. The mRNA–miRNA regulatory network was constructed by overlapping the target genes of differentially expressed miRNAs with the genes of differentially expressed mRNAs. STRING^56^ was applied to construct a protein–protein interaction network, Cytoscape was applied to fuse it with the mRNA–miRNA regulatory network and the fusion network was topologically analyzed using the CytoHubba plugin to screen the top 10 key nodes. Survival analysis of miR‐215‐5p expression in the TCGA database with overall survival time for DDL was implemented by LinkedOmics.^57^ miR‐215‐5p was predicted for regulatory relationship with MDM2 using TargetScan.

### 5.2 Cell lines and culture

The human liposarcoma cell line SW‐872 was purchased from the American Type Culture Collection (ATCC) and the cell line used in the validation experiment of short tandem repeat (STR) DNA profiling matched exactly with the SW‐872 cell line. SW‐872 cells were lysed and cultured in DMEM medium containing 10% fetal bovine serum at 37°C and 5% CO_2_. SW‐872 cells were transferred into a centrifuge tube containing 5 mL medium (DMEM + 15% FBS + 1% [penicillin–streptomycin solution] at a ratio of 1:3), centrifuges to collect cells, suspended with complete medium containing 10% fetal bovine serum, inoculated into a culture dish, gently blown and mixed well, and cultured at 37°C under 5% CO_2_ saturated humidity. When the cell density reached 80%, 1 mL 0.25% trypsin was added to the digest and collected the cells, which were passaged in a ratio of 1:3, and the culture was expanded at 37°C with 5% CO_2_ saturated humidity.

### 5.3 RT‐qPCR

miR‐215‐5p and MDM2 fluorescent real‐time quantitative PCR primers were designed by DNAMAN software (Table 3) and synthesized by Shanghai Saigon Co. Ltd. Cells were cultured in 96‐well plates at 2 × 10^5^ cells/well. 2 μL 5 × Prime Script Buffer, 0.5 μL Prime Script RT Enzyme Mix I, 0.5 μL Oligo (dT) 15 (15 μM), 0.5 μL Randommers (100 μM), Total RNA 500 ng, RNase Free dH_2_O were added to 10 μL, respectively, mixed well and briefly centrifuged, heated at 37°C for 15 min, 85°C for 5 s and diluted to 35 μL on a PCR instrument to obtain reverse transcribed cDNA. QPCR reaction: Forward primer 10 μM 0.4 μL, 0.4 μL Reverse primer 10 μM, 2XSYBR Select Master Mix (2 ×) 10 μL, RNase‐free water 4.8 μL, cDNA template 4 μL, 50 × ROX Reference Dye 20.4 μL, prepared into 20 μL system, mixed well, maintained on the instrument at 95°C, 10 min, 95°C, 15 s, 60°C 60 s, 95°C, 15 s, 40 cycles, melting curve 60°C 1 min, 95°C 15 s, 1 cycle, so as to obtain qPCR reaction results, using the 2‐ΔΔCt method for result quantification calculation. Three biological replicates were performed for each group.

**TABLE 3:** PCR primer sequences.

Gene	Primer	Sequence (5′–3′)	PCR Products
U6	Forward	CGCTTCGGCAGCACATATAC	
	Reverse	AAATATGGAACGCTTCACGA	
Homo GAPDH	Forward	TCAAGAAGGTGGTGAAGCAGG	115 bp
	Reverse	TCAAAGGTGGAGGAGTGGGT	
Homo MDM2	Forward	AGCAGGAATCATCGGACTCA	219 bp
	Reverse	TGTGGCGTTTTCTTTGTCGT	
miR‐215‐5p	loop primer	GTCGTATCCAGTGCAGGGTCCGAGGTATTCGCACTGGATACGACGTCTGTCA	
	F primer	TGCGCATGACCTATGAATTGA	

### 5.4 Dual luciferase reporter gene

Cells were cultured in 96‐well plates at 2 × 10^5^ cells/well. miR‐215‐5p or control was co‐transfected with pYr‐MirTarget‐MDM2 3 ′ UTR into cells according to the groups as indicated using lipofectamine 2000 as transfection reagent and Renilla specific firefly fluorescence intensity was calculated using Renilla luciferase as an internal reference after 48 h in the presence of firefly luciferase as an internal reference. Three biological replicates were performed for each group.

### 5.5 Cell phenotype experiments

The effect of miR‐215‐5p on cell proliferation activity was explored by MTT colorimetric assay: Cells were cultured in 96‐well plates at 2 × 10^5^ cells/well for 24, 48, and 72 h according to the groups, and 10 μL MTT was added to each well and cultured at 37°C for 4 h. The culture medium was aspirated and shaken with 150 μL DMSO for 10 min and the absorbance OD568 of each well was measured by a microplate reader. Three biological replicates were performed for each group.

Flow cytometry was used to investigate the effects of miR‐215‐5p on cell cycle and apoptosis: Cells in each group were seeded into 96‐well plates at 2 × 10^5^ cells/well, digested with 0.25% trypsin without EDTA, resuspended, washed, and fixed in 70% ethanol at 4°C for more than 4 h. Ribonuclease (RNase) was added and bathed in water at 37°C for 30 min; propidium iodide (PI) working solution was stained in the dark at 4°C for 30 min and then detected by flow cytometry. At the same time, the cells were taken according to the groups, digested with 0.25% trypsin without EDTA, resuspended, washed, and reacted using the AnnexinV‐APC/7‐AAD apoptosis detection kit according to the instructions and detected on the flow cytometer. Three biological replicates were performed for each group.

### 5.6 Cell scratch and invasion assay

Use a marker pen behind the 6‐hole plate, use a ruler to make comparison and evenly draw a horizontal line to pass through the hole at an interval of 0.5 cm. The treated cells, 5 × 10^5^ cells seeded in six‐well plates, were divided into groups, inoculated on a six‐well plate and cultured at 37°C and 5% CO_2_. When the cell density reached about 90%, a vertical scratch was made and photographs were taken at 0 and 48 h. Three biological replicates were performed for each group.

The treated cells, 3 × 10^5^ cells per group, were washed, resuspended, counted and diluted, inoculated in the prepared Matrigel‐transwell reaction system and cultured in 5% CO_2_ at 37°C for 24 h; the cells were fixed in 70% ice‐cold ethanol solution for 1 h, stained with crystal violet, and observed and photographed under a microscope.

A colony formation assay was performed to investigate the effect of miR‐215‐5p on colony formation: Dilute the cells according to the group, inoculate 300 cells in each group in a six‐well plate and seeded on six‐well plates at a density of 300 cells, and cultured for 3 weeks. When clones appeared, they were counted after methanol fixation and stained with crystal violet. Colony formation rate = colony count/inoculated cell count 100%. Three biological replicates were performed for each group.

### 5.7 Detection of MDM2 expression by immunofluorescence single labeling

Grouped cells were taken and seeded into 96‐well plates at 2 × 10^5^ cells/well. In the culture plate, the slides that had climbed the cells were immersed with PBS for 3 times, 3 min each time; the reptiles were fixed with 4% paraformaldehyde for 15 min, and the slides were immersed with PBS for 3 times, 3 min each time; 0.5% TritonX‐100 (prepared with PBS) was permeabilized at room temperature for 20 min; the slides were immersed with PBS for 3 times, PBS was blotted with absorbent paper, normal goat serum was added to the slides, and blocked at room temperature for 30 min; the blocking solution was absorbed with absorbent paper, without washing, a sufficient amount of diluted primary antibody (1:100) was added to each slide and placed in a wet box, and incubated overnight at 4°C; the reptiles were immersed with PBST for 3 times, 3 min each time, and the excess liquid on the reptiles was blotted with absorbent paper followed by dripping of diluted fluorescent secondary antibody (1:100), and the sections were immersed with PBST for 3 times, 3 min each time. Three biological replicates were performed for each group.

### 5.8 Detection of miR‐215‐5p expression by FISH

Grouped cells were taken and seeded into 96‐well plates at 2 × 10^5^ cells/well. Cell crawls were fixed in 4% paraformaldehyde (DEPC) for 20 min and washed three times by shaking on a detaining shaker in PBS (pH 7.4) for 5 min each. Gene strokes were circled, and proteinase K (20 μg/mL) was added dropwise for digestion for 6 min according to different index characteristics of different tissues. After rinsing with pure water, PBS was washed three times × 5 min. The prehybridization solution was dropped for 1 h in a 37°C incubator. The prehybridization solution was poured off, and the hybridization solution (containing probe miR‐215 at a concentration of 500 nM) was dropped and hybridized overnight at 42°C. The hybridization solution was washed off, 2 × SSC, 10 min at 37°C, 1 × SSC, 2 × 5 min at 37°C, and 10 min at 0.5 × SSC 37°C. If there are more non‐specific hybridizations, formamide washing can be added, and the hybridization solution containing secondary standard probes can be dropped and incubated at a dilution ratio of 1:400, 42°C for 3 h. The latter 2 × SSC, washed at 37°C for 10 min, 1 × SSC, washed at 37°C for 2 × 5 min, and 0.5 × SSC at 37°C for 10 min. Drop blocking solution: Drop blocking serum at room temperature for 30 min. Gutta mouse anti‐digoxigenin‐labeled peroxidase (anti‐DIG‐HRP) (Wuhan Boster Biological Engineering Co., Ltd. BA1051): The blocking solution was decanted and anti‐DIG‐HRP was added. Incubation was performed for 50 min at 37°C, followed by three × 5 min PBS washes. Dropping of CY3‐TSA: add CY3‐TSA reagent and allowing reacting at room temperature for 5 min, protected from light. After washing with PBS three times × 5 min. The sections were dropped with DAPI staining solution and incubated in the dark for 8 min, rinsed, and then dropped with anti‐fluorescence quenching mounting medium for mounting. Sections were observed and images were collected under a Nikon upright fluorescence microscope, and DAPI‐stained nuclei were blue with Cy3‐labeled red light under UV excitation. Three biological replicates were performed for each group.MiR‐215 probe:5’‐TCAATTCATAGGTCATTTTCCTGTATA.(TTTCATCATCAT ACATCATCAT) 30–3’. Secondary probe:5’‐DIG‐TTATGATGATGT ATGATGATGT‐3’.

### 5.9 CCK8 reaction

Grouped cells were taken and seeded into 96‐well plates at 2 × 10^5^ cells/well. RG7388 was added to SW‐872 mimics, and after 48 h of cell culture, 10 μL CCK8 was added to each well and cultured at 37°C for 4 h; the absorbance OD450 of each well was measured by a microplate reader. Three biological replicates were performed for each group.

### 5.10 WB was used to detect the expression changes of caspase‐3, MDM2, P53, P21, PCNA, Bax, and Bcl‐2

Grouped cells were taken and seeded into 96‐well plates at 2 × 10^5^ cells/well. After cell lysis, centrifugation and BCA quantification in different groups, the protein expression of caspase‐3 (Abcam Ab32351), MDM2 (Abcam ab259265), P53 (Abcam Ab1101), P21 (Abcam Ab109520), PCNA (Abcam Ab18197), Bax (Abcam Ab32503) and Bcl‐2 (Abcam Ab182858) was detected. METHODS: 5% stacking gel and 12% separation gel, sample denaturation and sample loading, electrophoresis and membrane transfer were prepared. The membrane was loaded into a 5% TBST blocking solution, prepared and shaken at room temperature for 2 h. Five milliliters of primary antibody diluent was added and kept at 4°C overnight. Ten milliliters of secondary antibody (1:1000 dilution) was placed on a shaker for 2 h. Color was developed and developed by compression exposure in a dark room. BandScan was used to analyze the gray value of the film, and the ratio of the intensity of the detected protein band to that of the protein band (%) was calculated, respectively. Three biological replicates were performed for each group.

### 5.11 Statistical analysis

In this study, SPSS 22.0 software was used for data processing. All data were expressed as mean ± standard deviation ($\bar{\chi }$ ± s). One‐way analysis of variance was performed for measurement data. The LDS method was used for pairwise comparison. The test level α = 0.05, *p* < 0.05 was considered statistically significant, and *p* < 0.01 was considered very significant.

## AUTHOR CONTRIBUTIONS

**zhengnan song:** Conceptualization (equal); data curation (equal); formal analysis (equal); funding acquisition (equal); software (equal); writing – original draft (lead); writing – review and editing (lead). **Jingping Bai:** Conceptualization (equal); funding acquisition (equal); investigation (equal); methodology (equal); project administration (equal); resources (equal); supervision (equal); validation (equal); visualization (equal). **Renbing Jiang:** Data curation (equal); resources (equal). **Junshen Wu:** Software (equal); supervision (equal); validation (equal); visualization (equal). **Wenpeng Yang:** Software (equal); validation (equal); visualization (equal).

## CONFLICT OF INTEREST STATEMENT

The authors do not have any potential conflicts of interest.

## Supporting information


**Supplementary 1A.** GO functional analysis
**Supplementary 1B**. KEGG functional analysis

## Data Availability

The data that support the findings of this study are available in GEO at (www.ncbi.nlm.nih.gov/geo/), reference number GSE21122 and GSE36982. These data were derived from the following resources available in the public domain: GSE21122 (https://www.ncbi.nlm.nih.gov/geo/query/acc.cgi?acc=GSE21122)GSE36982 (https://www.ncbi.nlm.nih.gov/geo/query/acc.cgi?acc=GSE36982). The data that support the findings of this study are available in the supplementary material of this article.
