# Supplementary material for: MicroRNA‐215‐5p promotes proliferation, invasion, and inhibits apoptosis in liposarcoma cells by targeting MDM2
Source: Cancer Med. 2023 May 3;12(12):13455–70. doi: 10.1002/cam4.5993 (PMC10315806; doi:10.1002/cam4.5993)
Supplement: Supplementary file 1 — Supplementary 1A. GO functional analysis Supplementary 1B. KEGG functional analysis [file CAM4-12-13455-s001.doc]

Supplement 1A. GO functional analysis

| Category | Term | Genes | FDR |
| --- | --- | --- | --- |
| GOTERM_BP_DIRECT | GO:0071300~cellular response to retinoic acid | COL1A1, FZD4, MYC, PPARG, PCK1 | 0.040825 |
| GOTERM_BP_DIRECT | GO:0071364~cellular response to epidermal growth factor stimulus | COL1A1, MYC, ID1, SNAI2 | 0.046183 |

Supplement 1B. KEGG functional analysis

| Category | Term | Count | Genes | FDR |
| --- | --- | --- | --- | --- |
| hsa04152 | AMPK signaling pathway | 5 | PPARG,PCK1,SCD,IRS2,PFKFB3 | 0.0023 |
| hsa04923 | Regulation of lipolysis in adipocytes | 4 | PLA2G16,PTGER3,IRS2,ABHD5 | 0.0023 |
| hsa04550 | Signaling pathways regulating pluripotency of stem cells | 4 | ID1,ID4,FZD4,MYC | 0.0209 |
| hsa05200 | Pathways in cancer | 7 | PPARG,FOS,PTGER3,CKS2,CDKN2A,FZD4,MYC | 0.0209 |
| hsa04390 | Hippo signaling pathway | 4 | ID1,SNAI2,FZD4,MYC | 0.0226 |
| hsa03320 | PPAR signaling pathway | 3 | PPARG,PCK1,SCD | 0.0252 |
| hsa04350 | TGF-beta signaling pathway | 3 | ID1,ID4,MYC | 0.0321 |
| hsa01040 | Biosynthesis of unsaturated fatty acids | 2 | ACOT2,SCD | 0.0363 |
| hsa00062 | Fatty acid elongation | 2 | ACOT2,HADH | 0.0376 |
